# Studying the Complex Formation of Sulfonatocalix[4]naphthalene and Meloxicam towards Enhancing Its Solubility and Dissolution Performance

**DOI:** 10.3390/pharmaceutics13070994

**Published:** 2021-06-30

**Authors:** Tayel A. Al Hujran, Mousa K. Magharbeh, Samer Al-Gharabli, Rula R. Haddadin, Manal N. Al Soub, Hesham M. Tawfeek

**Affiliations:** 1 The Department of Pharmaceutical Chemistry, Faculty of Pharmacy, Mutah University, Al-Karak 61710, Jordan; Magharbeh@mutah.edu.jo (M.K.M.); rola.r.haddadin@hotmail.com (R.R.H.); dmanal19@yahoo.com (M.N.A.S.); 2 Pharmaceutical and Chemical Engineering Department, School of Applied Medical Sciences, German Jordanian University, Amman 11118, Jordan; samer.gharabli@gju.edu.jo; 3 Department of Industrial Pharmacy, Faculty of Pharmacy, Assiut University, Assiut 71515, Egypt; Heshamtawfeek@aun.edu.eg

**Keywords:** meloxicam, dissolution, inclusion complexes, phase solubility, sulfonatocalix[4]naphthalene, physicochemical characterization

## Abstract

The interaction between meloxicam and sulfonatocalix[4]naphthalene was investigated to improve the meloxicam solubility and its dissolution performance. Solubility behavior was investigated in distilled water (DW) and at different pH conditions. Besides, solid systems were prepared in a 1:1 molar ratio using coevaporate, kneading, and simple physical mixture techniques. Further, they were characterized by PXRD, FT-IR, DCS, and TGA. In vitro dissolution rate for coevaporate, kneaded, and physical mixture powders were also investigated. Solubility study revealed that meloxicam solubility significantly increased about 23.99 folds at phosphate buffer of pH 7.4 in the presence of sulfonatocalix[4]naphthalene. The solubility phase diagram was classified as A_L_ type, indicating the formation of 1:1 stoichiometric inclusion complex. PXRD, FT-IR, DCS, and TGA pointed out the formation of an inclusion complex between meloxicam and sulfonatocalix[4]naphthalene solid powders prepared using coevaporate technique. In addition, in vitro meloxicam dissolution studies revealed an improvement of the drug dissolution rate. Furthermore, a significantly higher drug release (*p* ≤ 0.05) and a complete dissolution was achieved during the first 10 min compared with the other solid powders and commercial meloxicam product. The coevaporate product has the highest increasing dissolution fold and RDR_10_ in the investigated media, with average values ranging from 5.4–65.28 folds and 7.3–90.7, respectively. In conclusion, sulfonatocalix[4]naphthalene is a promising host carrier for enhancing the solubility and dissolution performance of meloxicam with an anticipated enhanced bioavailability and fast action for acute and chronic pain disorders.

## 1. Introduction

The supramolecular chemistry showed a broad interest during the last few decades in different aspects of applications such as electrochemical sensors, catalysis, drug delivery systems, biotherapy, and self-healing [1,2,3,4]. New macrocycles were synthesized such as crown ethers [5], calix[*n*]arenes [6], etc., which have the ability to host different types of molecules for numerous applications [6].

Calix[*n*]arenes are cyclic oligomers that have different sizes depending on the number of the repeated phenolic unit, ranging from 4 to 8 units [7,8,9,10]. This type of macrocyclic has a well-defined deep hydrophobic cavity surrounded by a hydrophobic upper rim and hydrophilic lower rim. Water-soluble calixarenes were synthesized by condensation of *p*-*tert*-butylphenol with formaldehyde [7]. Further sulfonic groups were introduced at the para position of the repeated phenol unit to give water-soluble *p*-sulfonic calix[*n*]arenes [6] or by modification the lower rim like preparation of water-soluble *O*-phosphonate calix[*n*]arenes [11].

The cyclic oligosaccharide cyclodextrins (CDs) and their CDs different chemical synthesis derivatives such as hydroxylpropyl-*β*-CD and methyl-*β* -CD are the most usable carriers in the drug delivery [12,13,14]. On other hand, many studies reported inclusion complexes of *para*-sulfonatocalix[*n*]arenes [15,16,17] or *O*-phosphonate calix[*n*]arenes with different drugs, such as carbamazepine [18] mycophenolate [19] mofetil, Carvedilol [19], nifedipine [11,18], niclosamide [11,18], and furosemide [15,18]. In terms of toxicity, *Para*- sulfonatocalix[*n*]arenes showed very low toxicity and better compatibility compared with that of other macrocylclics [20,21], hence, they could be used for drug delivery applications. Furthermore, the sulfonated calix[*n*]arenes have a strong hydrophilic upper rim and hydrophobic inner cavity due to the sulfonate groups and the aromatic rings, which are connected by –CH_2−_ linkage, respectively. By contrast, cyclodextrins’ inner cavity is lined by the ether linkage, which is contacted the glucopyranose units [22,23].

**Scheme 1:**
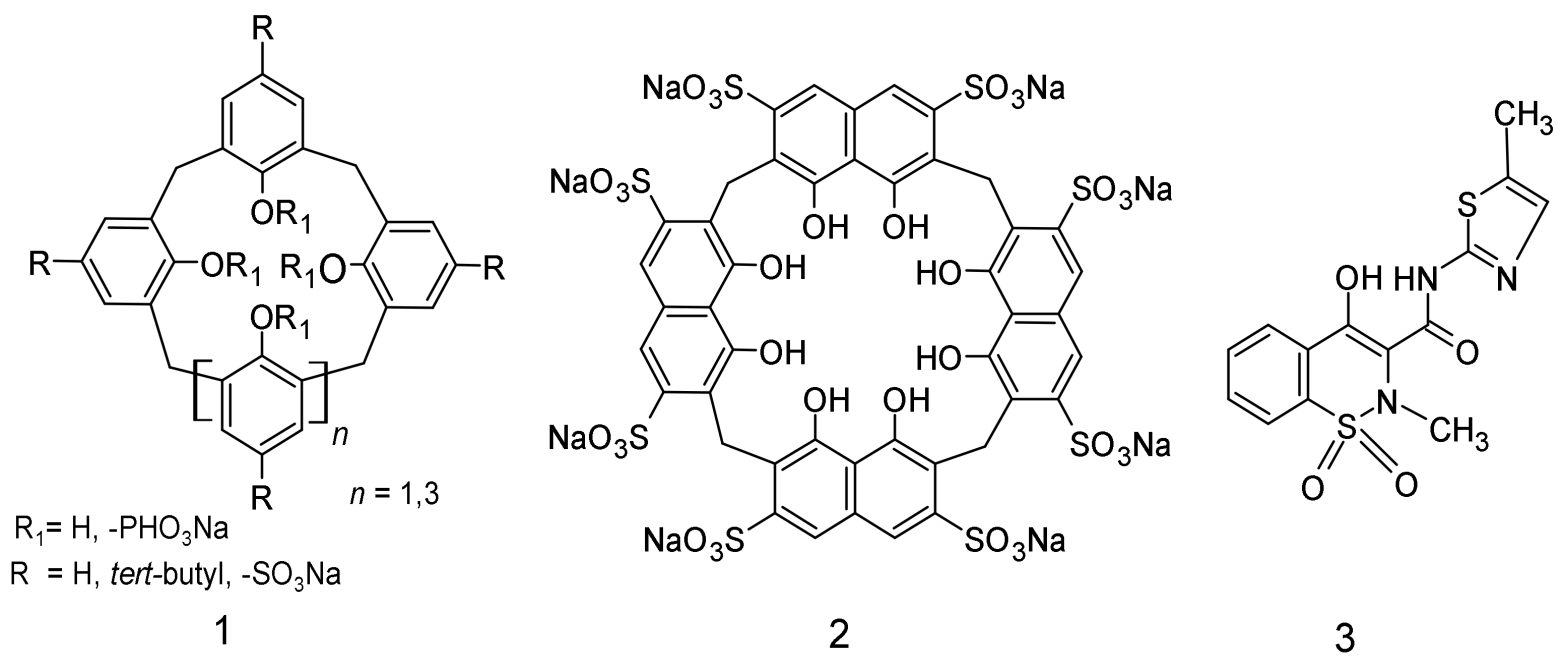
Structures of *p*-sulfonic or *O*-phosphonate calix[*n*]arenes (1), sulfonatocalix[4]naphthalene (2), and meloxicam (3).

The most water-soluble derivative is the sulfonatocalix[4]naphthalene (as illustrated in Scheme 1, compound **2**) which was prepared in 1989 by Poh’s group (as illustrated in Scheme 1) [24]. It was obtained from the reaction of chromotropic acid disodium salt with an excess amount of formaldehyde in an aqueous solution. This water-soluble calix[*n*]arene is considered a cyclodextrin analog [4]. Since then, Poh’s group reported several supramolecular complexation studies in an aqueous solution. Furthermore, they concluded that this highly water-soluble derivative showed typical host–guest properties with various guest compounds [25,26]. Besides, the higher solubility in water, sulfonatocalix[4]naphthalene can host the different three types of the cyclodextrins (*α*-, *β*-, and *γ*-) in water [27].

Meloxicam (ME) (as illustrated in Scheme 1, compound **3**) is a nonsteroidal anti-inflammatory drug (NSAIDs), which is still one of the most widely prescribed medications to treat joint diseases such as rheumatoid arthritis, osteoarthritis, and other musculoskeletal disorders [28,29,30,31,32]. It has poor aqueous solubility and higher permeability, which fall under class II medications in biopharmaceutical classes systems. However, the poorly aqueous solubility and wettability of ME resulted in a slower dissolution rate of the drug, and hence, a lower oral bioavailability concomitant with slowing its onset of action [33,34]. Enhancing the aqueous solubility of ME could facilitate its oral absorption and bioavailability and reduce its onset of action for the treatment of different types of acute pain [34,35].

Drugs that are classified by biopharmaceutical classification as type-II and IV, poorly soluble drugs, still have great issues in their formulation and efficiency after oral administration. Many techniques were adapted to address this point and enhance the solubility of these compounds, such as using solid dispersions [36], hydrophilic carriers [37], incorporation into lipid vesicles [38], micronization [39], cocrystals [34], adsorption [40], and complexation [41,42].

In the present work, the interaction between ME and sulfonatocalix[4]naphthalene was studied to enhance the solubility and the dissolution performance of the drug. Complex behavior in solution was investigated, as well as in the solid-state. A phase solubility diagram was determined, and the prepared solid systems were characterized by performing XRD, FTIR, TGA, and DSC studies. Furthermore, in vitro dissolution performance was also investigated in different media at pH values of 7.4, 1.2, and in distilled water.

## 2. Materials and Methods

### 2.1. Materials

Meloxicam was kindly donated by Dar Adawa Company-Jordan (Amman). Chromotropic acid disodium salt dehydrate (Sigma–Aldrich, Hamburg, Germany), methanol (Fischer, Loughborough, UK), acetone (Tedia–Fairfield, OH, USA), hydrochloric acid, potassium dihydrogen (AZ Chem. for chemicals, Manchester, UK), and dipotassium hydrogen phosphate (PRS, Panreac–Espana, Barcelona, Spain).

### 2.2. Methods

#### 2.2.1. Phase Solubility Studies

The sulfonatocalix[4]naphthalene was prepared according to the reported literature procedure [24] and used in the following experiments. Solubility studies were carried out according to the method reported by Higuchi and Connors [43]. ME in amounts that exceeded its solubility (16 mM) was added to 5 mL of distilled water (DW) and at different pH solutions 1.2 and 7.4, which resembles the stomach and intestinal pH values, containing increasing concentrations of sulfonatocalix[4]naphthalene (3.2–16 mM). This performing the following ME: sulfonatocalix[4]naphthalene molar ratios of 1:0, 1:0.2, 1:0.4, 1:0.6, 1:0.8, 1:1. At least three samples of each molar ratio were prepared. The sealed glass vials were shaken for 4 days at 25 ± 0.5 °C, after which equilibrium was reached [42]. The vial's contents were subjected to centrifugation (Boeco–Germany, Hamburg, Germany) at 3000 rpm for 20 min. The supernatant was collected and filtered by using a 0.45 µm syringe filter (Syringe Filter PTFE, Santa Cruz Biotechnology, Inc., Dallas, TX, USA) and appropriately diluted. A portion of the sample was analyzed using UV/VIS double beam spectrophotometer SCOTech SPUV-26 (Dingelstadt, Hamburg, Germany) at λ_max_ of 360 nm against blanks prepared in the same concentration of sulfonatocalix[4]naphthalene at three different types of the investigated media to cancel any absorbance that may be exhibited by the sulfonatocalix[4]naphthalene. The apparent stability constant (*Ks*) of the prepared complexes was calculated from the *slope* of the obtained phase-solubility diagrams using the following equation [44].

*Ks* = *slope*/*S*_o_(1 − *slope*) (1)

The *slope* is obtained from the initial straight-line portion of the plot of ME concentration against sulfonatocalix[4]naphthalene concentration and *S_o_* is the solubility of ME alone in the different investigated media. All solubility measurements were performed in triplicate and taken as a mean ± S.D.

#### 2.2.2. Preparation of Inclusion Complexes

Solid complexes of ME and sulfonatocalix[4]naphthalene were performed by different techniques, which are described below. Based on the results obtained from phase solubility studies, the molar ratio between ME and host molecule was kept at 1:1 in the prepared systems. Sulfonatocalix[4]naphthalene was prepared according to the reported literature procedure [24]. 

##### Physical Mixture

The physical mixture was prepared by homogeneous blending of previously sieved powders through mesh no. (150 μm) of equal molar ratio (1:1) of ME and sulfonatocalix[4]naphthalene. Blending was performed by using a spatula and mortar for 15 min, and then, the obtained powder was stored in a desiccator over P_2_O_5_ till further analysis.

##### Kneading

The kneaded mixture was obtained by adding few drops of water to the sulfonatocalix[4]naphthalene in a mortar and mixing to obtain a homogeneous paste. Then, ME was added slowly and the mixture was kneaded for further 15 min, during the process few drops of water were added. The resulting paste was dried in a vacuum oven (Thermo Stable OV-30, Gangwon-do, Korea) at 40 °C for 24 h. The resulting powder was sieved through mesh no. 150 μm and stored in a desiccator over P_2_O_5_ till further analysis.

##### Co-Evaporation

An aqueous solution of sulfonatocalix[4]naphthalene in 20 mL distilled water was added to 5 mL acetone containing the calculated amounts of ME. The mixture was stirred for 1 h at room temperature. Then, the solvent was removed by using a rotary evaporator (Buchi–Switzerland) at 45 ± 0.5 °C, followed by drying in a vacuum oven (Thermo Stable OV-30, Wonju-Shi, Korea) at 40 °C for 24 h. The obtained solid was pulverized into a fine powder, sieved through mesh no. 150 μm, and stored as previously mentioned. 

#### 2.2.3. Characterization of Solid Systems

##### Differential Scanning Calorimetry (DSC)

DSC thermograms were obtained by using a Shimadzu DSC-50 (Tokyo, Japan). Approximately 2–5 mg powdered samples were placed in fifty microliter aluminum pans with 0.1 mm thickness. Pans were then sealed with an aluminum cover of 0.1 mm thickness and an empty pan was used as a reference. DSC thermograms were recorded by application of heating rate of 10 °C min^−1^ and temperature from room temperature to 650 °C under nitrogen flow of 40 mL/min. DSC temperature was calibrated using indium. DSC gives information about the changes in the energy of the materials (specific heat capacity and enthalpy), as well as allows identifying the endo and exoenergic processes.

##### Thermogravimetric Analysis (TGA), and Derivative Thermogravimetry (DTG) 

The thermal properties of the physical, kneaded, and coevaporate mixture samples were evaluated using thermosanalyzer Jupiter STA 449 F5 (Netzsch, Germany). Thermogravimetric analysis (TGA), derivative thermogravimetry (DTG), were accomplished over a temperature range of 25–1100 °C. Sample were placed in the aluminum oxid crucible (Al_2_O_3_) and heated from 25 to 1100 °C with a heating rate of 20 °C/min under a nitrogen atmosphere. TGA and DTG were used for the thermal stability characterization of the investigated samples. The obtained curves were analyzed using the Netzsch Proteus Analysis Software.

##### Powder X-ray Diffraction (PXRD)

The powder X-ray diffraction patterns of sulfonatocalix[4]naphthalene, ME, and their solid complexes were recorded using a Philips 1710 powder diffractometer with Cu K*α* radiation (1.54056 Å). A Cu target tube operated at a voltage of 40 kV operating with 40 mA current and a single crystal graphite monochromator were employed. 0.6°/min was set as scanning speed and wide-angle diffraction of 4° < 2θ < 60° were adapted. XRD instrument was calibrated using powder of polycrystalline silicon standard.

##### Fourier-Transform Infrared Spectroscopy (FT-IR)

Attenuated total reflection–Fourier transform infrared spectroscopy (ATR-FTIR) was used to assess physicochemical interaction of the physical, kneaded, and coevaporated product samples. Data were recorded using Bruker Vertex 80 v ATR-FTIR apparatus. A 512 scan with a resolution of 4 cm^−1^ was used during the data collection.

#### 2.2.4. Dissolution Studies

In vitro dissolution behaviors of the ME and sulfonatocalix[4]naphthalene and their respective solid systems were carried out in 500 mL dissolution medium of different pH conditions, pH 1.2, phosphate buffer of pH 7.4, and distilled water (DW) using USP type-II (Paddle) in a dissolution tester (Pharma test PTWS 820D, Hainburg, Germany). Samples equivalent to 15 mg of ME were sprinkled in the dissolution medium using the stirring speed of 100 ± 1.0 rpm and temperature of 37 ± 0.5 °C. A 5 mL aliquot was withdrawn at 5; 10; 15; 25; 35; 45; 60; 90 min, filtered through 0.45 µm filters (Syringe Filter PTFE, Santa Cruz Biotechnology, Inc., Dallas, TX, USA) and replaced with 5 mL of fresh dissolution medium. The filtered samples were suitably diluted whenever necessary and analyzed by UV spectrophotometer at λ_max_ of 360 nm. Dissolution experiments were carried out in triplicate for all the prepared samples and the mean values were taken ±S.D.

#### 2.2.5. Statistical Analysis

The dissolution rate parameters were estimated using one–way analysis of variance (ANOVA) by using software (SPSS version 25), followed by Scheffe’s multiple comparisons test. The statistical analysis results were considered significant if the *p*-value ≤ 0.05. All values are expressed as their mean ± standard deviation.

## 3. Results and Discussion

### 3.1. Phase Solubility Studies

**Figure 1:**
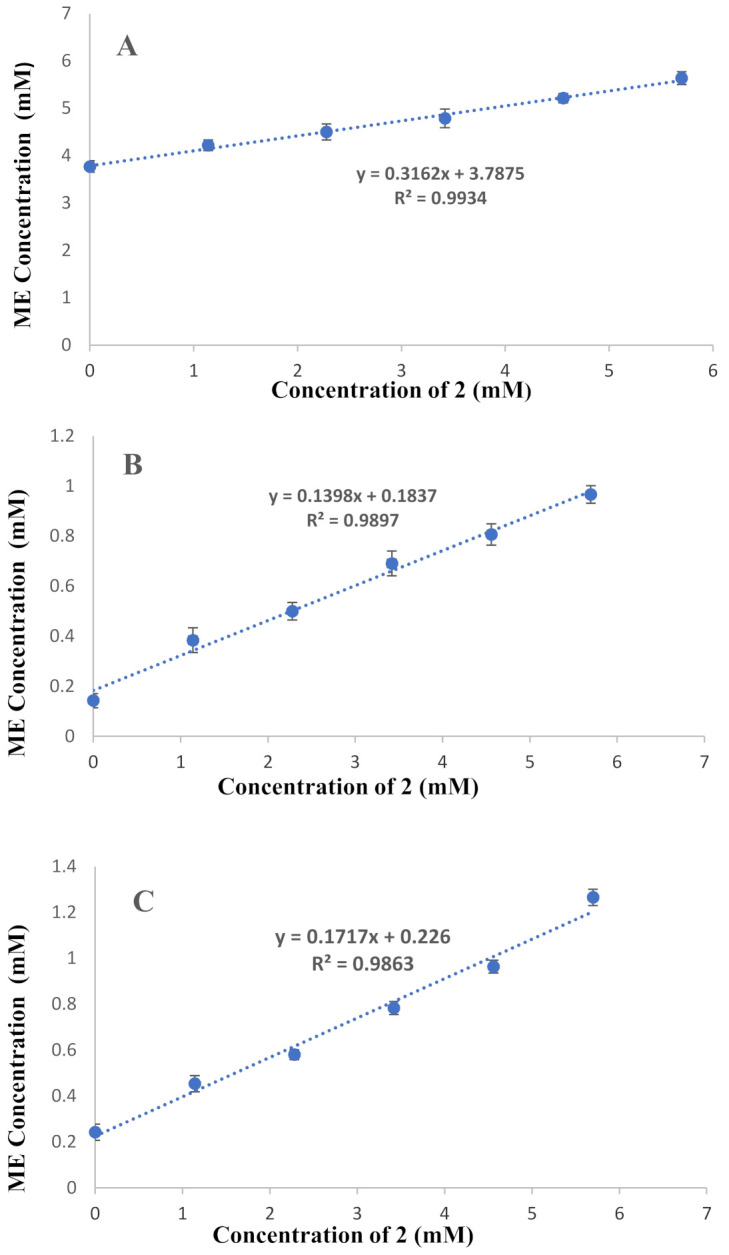
Phase solubility diagram of ME in presence of different concentrations of sulfonatocalix[4]naphthalene at temp. 25.5 ± 0.5 °C. (**A**) Phosphate buffer of pH 7.4, (**B**) pH 1.2, and (**C**) distilled water.

The oral bioavailability of ME is very poor due to poor solubility in an aqueous solution, making the solubility and dissolution are rate-determining steps in the absorption of the drug [45]. The result of this study showed that sulfonatocalix[4]naphthalene could be the host of ME in a 1:1 molar ratio in their deep hydrophobic cavity. Hence, a significant increase in the solubility, and accordingly, the dissolution rate, of ME was observed. Phase solubility diagram of ME at 25 ± 0.5 °C in the presence of increasing concentrations of sulfonatocalix[4]naphthalene was obtained by plotting equilibrium concentrations of the drug against sulfonatocalix[4]naphthalene concentrations as shown in Figure 1, according to the method reported by Higuchi and Connors [43]. The phase solubility of ME in the aqueous solution of sulfonatocalix[4]naphthalene at different pH solutions showed a linear pattern in enhancing the solubility of ME as the concentration of sulfonatocalix[4]naphthalene increased compared with that of the drug alone. The constructed phase solubility curves could be classified as A_L_ type, which suggested the formation of the 1:1 water-soluble ME-sulfonatocalix[4]naphthalene inclusion complex. This result might be attributed to the weak interaction forces including hydrogen bonding, *π-π* interactions, dipole-dipole interaction, or electrostatic interaction between hydrophobic cavity or hydroxyl groups of sulfonatecalix[4]naphthalene and drug aromatic rings, or other functional groups of the drug molecules [15,16,46]. The calculated stability constants (Ks) of ME-sulfonatocalix[4]naphthalene complex (1:1) at 25 ± 0.5 °C are presented in Table 1. The stability constants were calculated from the linear regression analysis of the obtained phase solubility diagram and were found to be 1159.739 ± 0.084, 653.256 ± 0.167, and 555.953 ± 0.021 M^−1^ for phosphate buffer, distilled water, and pH 1.2, respectively. The largest and the smallest stability constants were observed at phosphate buffer pH 7.4 and at pH 1.2, as depicted in Table 1. This result could be attributed to the weak acidity nature of the ME which formed a zwitterionic compound with two lower pKa values 1.09 and 4.18 and the solubility in both around 0.6 µg/mL [33]. For that, ME has a higher solubility in basic conditions and low solubility in acidic media. This result also in agreement with the reported results found by Jin et al. [28] On the other hand, the formation of the ME- sulfonatocalix[4]naphthalene complex in an aqueous medium led to enhancing its water solubility in acidic and basic media which will enhance its dissolution and presumably its bioavailability, and hence, faster action can be performed.

### 3.2. Thermal Analysis

#### 3.2.1. DSC

**Figure 2:**
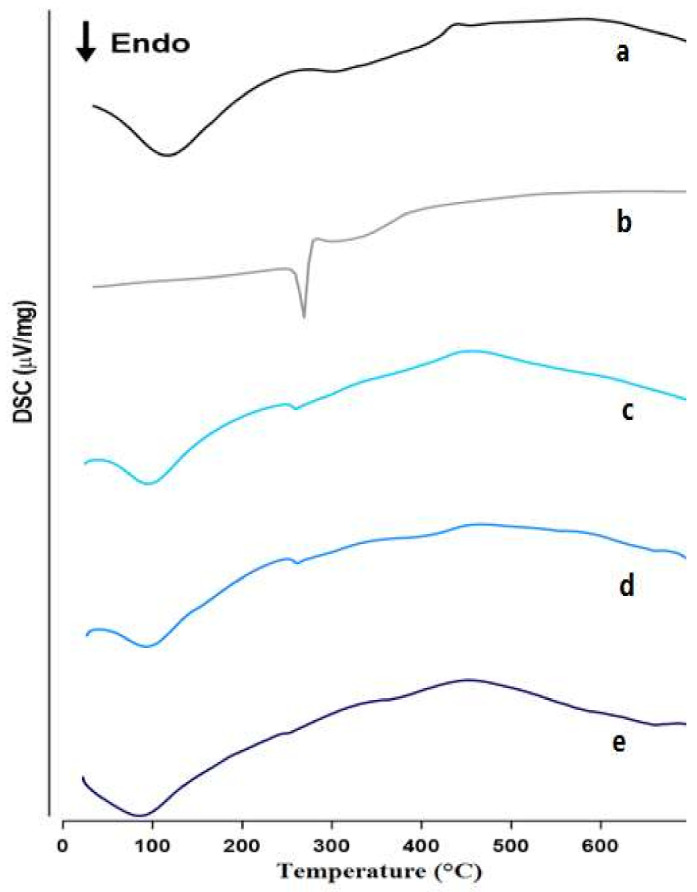
DSC thermograms of following: (**a**) sulfonatocalix[4]naphthalene (C), (**b**) meloxicam (ME), (**c**) physical mixture (PM), (**d**) kneaded (KN), and (**e**) coevaporate (CO) powders.

Thermal analysis is an important method for the recognition and characterization the formation of complexes between drugs and their carrier [47]. DSC is the most commonly employed technique to assess the number of amorphous phases existing in systems containing more than one component [48] and a valuable method for detecting drugs interaction and compatibility with excipient [49,50]. Therefore, the thermal behavior of ME, sulfonatocalix[4]naphthalene, solid complexes, and physical mixture were studied using DSC to characterize the possibility of complexation and/or any possible interaction. The DSC thermograms of sulfonatocalix[4]naphthalene, ME alone, and respective solid systems are shown in Figure 2. The DSC thermogram of ME alone, Figure 2, showed one main characteristic sharp endothermic melting peak at around 256 °C, which corresponds to its melting point [51]. Sulfonatocalix[4]naphthalene showed two major endothermic peaks, the first attributed to sampling dehydration at 100 °C and the second peak refers to fusion or decomposition at 750 °C. The DSC thermograms of the physical and kneaded mixtures of the drug revealed that the intensity of the endothermic melting point peak of ME was highly decreased and shifted to the lower temperature around 245 °C and 243 °C, respectively. However, in the case of a coevaporate mixture, the complete disappearance of drug melting endotherm was observed. These results revealed some sort of molecular interaction between ME and sulfonatocalix[4]naphthalene. Much more information about the type of interaction with the possibility of complex formation as well as the conversion of ME from crystalline to an amorphous or partially amorphous structure will be investigated from XRD, FT-IR, and other performed studies.

#### 3.2.2. TGA

**Figure 3:**
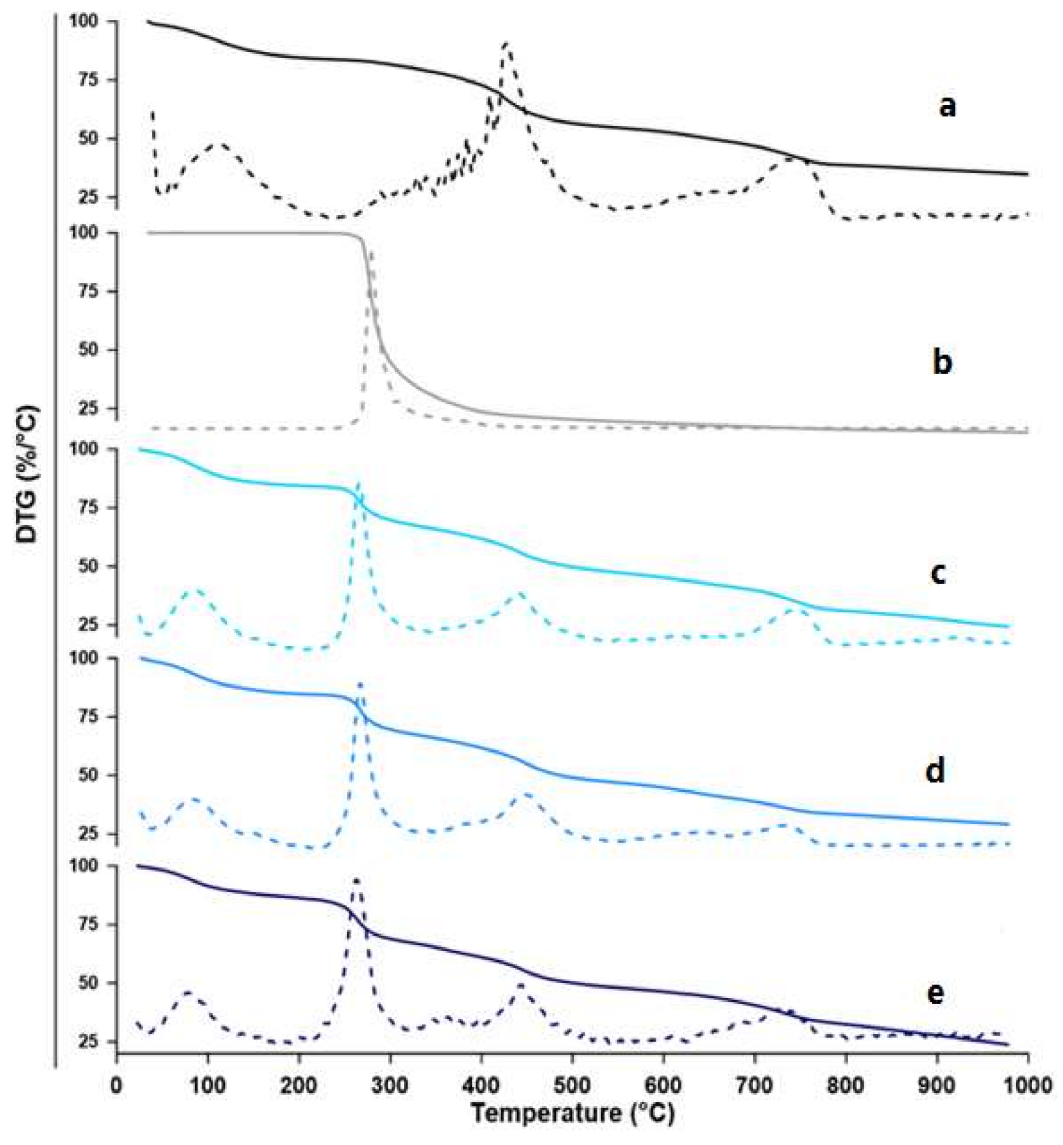
TGA/DTG curves of following: (**a**) sulfonatocalix[4]naphthalene (C), (**b**) meloxicam (ME), (**c**) physical mixture (PM), (**d**) kneaded (KN), and (**e**) coevaporate (CO) powders.

Thermogravimetric Analysis (TGA) is an important and widely accepted tool used to distinguish all types of amorphous materials, such as drug inclusion complexes and polymeric materials [47,52]. The thermal stability of physical, kneaded, and coevaporate solid systems of ME and sulfonatocalix[4]naphthalene was investigated using TGA as shown in Figure 3. The TGA curves analysis showed that ME starts its degradation at 277.5 °C in the single exothermic event, while sulfonatocalix[4]naphthalene showed the beginning of degradation at 424.2 °C in two exothermic events and losing water at a range of 61–157 °C. The derivatograms showed that the decomposition of the physical mixture, kneaded, and coevaporate products have three major decomposition patterns (as illustrated in Figure 3). The initial decomposition temperature, which occurred due to dehydration, appears from 50 up to 130 °C, while the second step occurred between 230 °C and 300 °C. The temperature values for the 50-weight percentage decomposition increased up until 450 °C. The TGA result showed the degradations occurred at lower temperature compared with the ME alone. Also, the TGA showed the weight loss peaks of the physical mixture, kneaded, and coevaporate products shifted to 262 °C, 260 °C, and 254 °C, respectively, compared with losing weight peak of the pure ME, which observed at 277.5 °C. On the other hand, coevaporate product shows the lowest shifted losing water peak position compared with those observed for peaks of the physical mixture, kneaded products, and sulfonatocalix[4]naphthalene alone. This result indicated the formation of an inclusion complex between ME and sulfonatocalix[4]naphthalene and/or formation of highly amorphous material in coevaporation product. Other researchers reported similar findings; they concluded that the shifting weight loss peaks to lower position to the formation of an inclusion complex with the investigated host molecule [53,54].

#### 3.2.3. Powder X-ray Diffractometry (PXRD)

**Figure 4:**
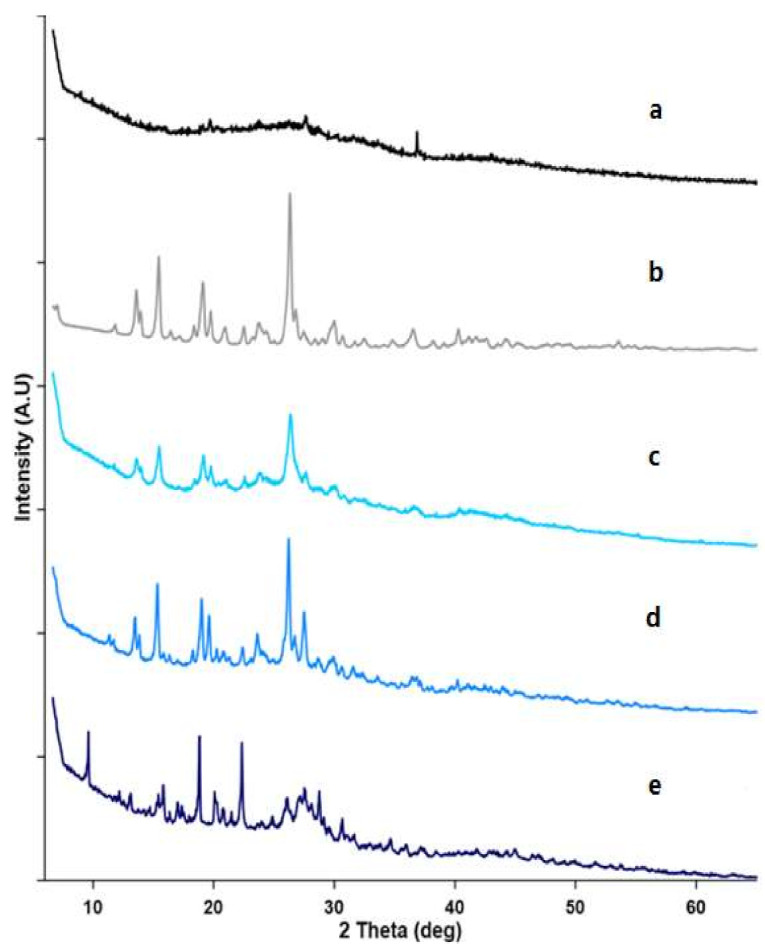
PXRD of following: (**a**) sulfonatocalix[4]naphthalene (C), (**b**) meloxicam (ME), (**c**) physical mixture (PM), (**d**) kneaded (KN), and (**e**) coevaporate (CO) powders.

The PXRD is performed to get much more evidence about the interaction between ME and sulfonatocalix[4]naphthalene. The PXRD pattern of ME, sulfonatocalix[4]naphthalene, and their corresponding 1:1 molar ratio powdered systems are shown in Figure 4. The X-ray diffraction of the sulfonatocalix[4]naphthalene did not show diffraction peaks indicating its amorphous pattern (as illustrated in Figure 4, trace a). On the contrary, ME showed several sharp high-intensity peaks values at 2θ diffraction angle of 13.2 (1022), 15.0 (1477), 18.8 (1136), 26.2 (2431), 30.1 (568), and 36.3 (477), indicating its crystalline nature (as illustrated in Figure 4, trace b) [55]. Physical and kneaded solid systems still reflected the crystalline peaks of ME; however, they were reduced in their intensity and became broader. Thus, this feature delineates the incomplete complex formation between sulfonatocalix[4]naphthalene and ME, which could possibly be attributed to the method and/or the low amount of sulfonatocalix[4]naphthalene in the prepared systems. However, the diffraction peaks of ME completely disappeared in the coevaporate product concomitant with the appearance of new XRD sharp peaks at 2θ diffraction angle at position 10.0 (363), 19.1 (613), and 23.8 (590). This behavior could be possibly attributed to the formation of an inclusion complex between the ME and sulfonatocalix[4]naphthalene and/or the formation of a new complex compound with some crystallinity. Similar findings were also reported by other researchers, who concluded that the formation of new sharp peaks in XRD confirmed the formation of an inclusion complex with the investigated host molecule [56,57]. In addition, these results agreed with DSC result, which confirmed the disappearance of characteristic melting endotherm of ME observed in the coevaporate product. Conclusively, XRD data confirmed the formation of an inclusion complex.

#### 3.2.4. Infrared Spectroscopy

**Figure 5:**
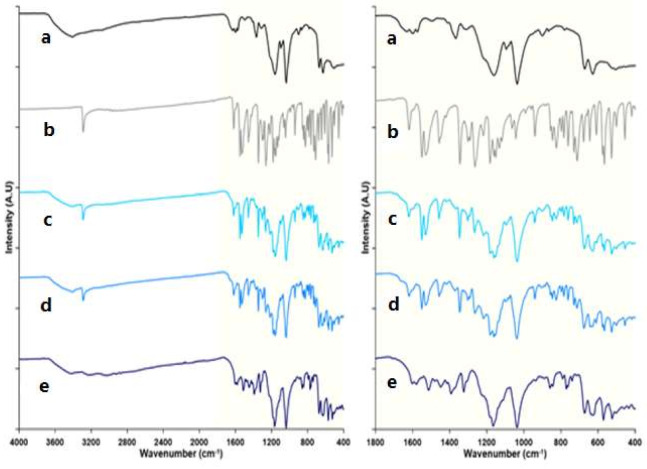
FTIR spectra of following: (**a**) sulfonatocalix[4]naphthalene (C), (**b**) meloxicam (ME), (**c**) physical mixture (PM), (**d**) kneaded (KN), and (**e**) coevaporate (CO) powders.

Figure 5 shows FT-IR spectra of ME, sulfonatocalix[4]naphthalene, and their solid systems formed by physical mixtures, kneading, and coevaporate techniques. A sulfonatocalix[4]naphthalene spectrum demonstrated a broad peak in the region of 2900–3500 cm^−1^, resulting from vibration stretching of hydroxyl groups; other peaks were in the range of 1631 to 1571 cm^−1^, stretching the double bond in naphthalene aromatic rings and a shorter stretching double band at 1381 cm^−1^, a large region which displays distinct peaks in the region of 1161 to 500 cm^−1^ (as illustrated in Figure 5, trace a). FT-IR spectrum of ME showed sharp characteristic peaks, due to the stretching vibration of the amid, carbonyl, aromatic ring double bond, and two sulphonyl groups, at 3273, 1691, 1452, 1345, and 1155 cm^−1^, respectively (as illustrated in Figure 5, trace b). The FT-IR spectra of the physical mixture and the kneaded solid system showed a reduced and broad drug amid band. On the other hand, the characteristic bands of the carbonyl, aromatic ring double bond, and two sulphonyl groups were reduced in their intensity and shifted to 1595, 1440, 1333, and 1036 cm^−1^, respectively, for physical mixture, in comparison with the original peak in pure ME. This observation could be attributed to the possibility of intermolecular hydrogen bonding between the amide carbonyl and sulphonyl groups of the drug and hydroxyl group (lower rim) of the sulfonatocalix[4]naphthalene or π-π interaction between the aromatic rings of the drug and the sulfonatocalix[4]naphthalene cavity. Whereas in FT-IR spectrum of coevaporate product, the characteristic bands of the drug groups disappeared. Furthermore, the characteristic bands of the sulfonatocalix[4]naphthalene in coevaporate product also shifted to a new positions 1607, 1159, 1524 cm^−1^ compared to that of pure sulfonatocalix[4]naphthalene, while the rest of the drug characteristic bands were reduced in intensity. Such behavior supported the strong interaction with inclusion complexation inside the cavity of sulfonatocalix[4]naphthalene.

### 3.3. Dissolution Studies

**Figure 6:**
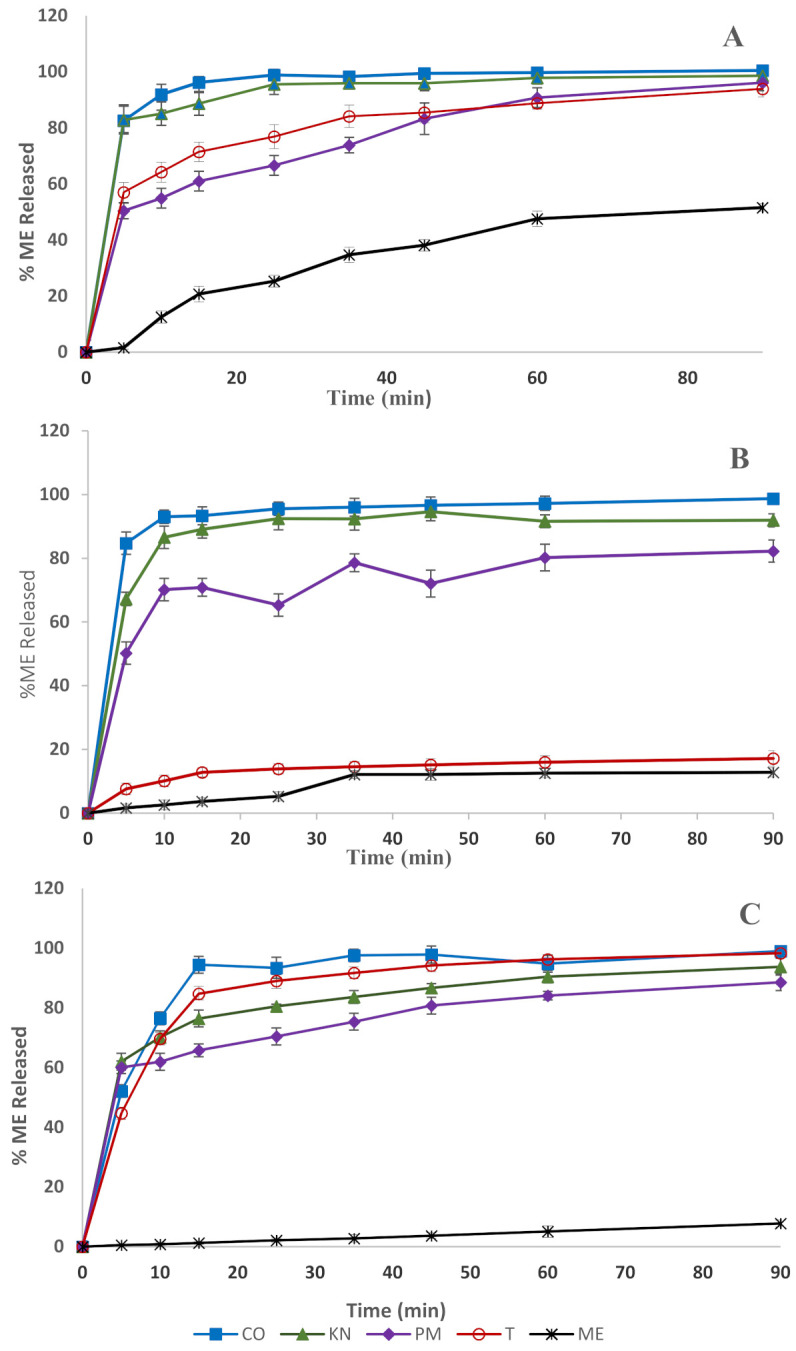
In vitro dissolution performance of ME from different prepared systems; coevaporate (CO), kneaded (KN), physical mixture (PM), commercial ME (T), and ME untreated powder (ME); in (**A**) Phosphate buffer pH 7.4; (**B**) pH 1.2; and (**C**) distilled water; Key: Co (filled square), KN (filled triangle), PM (filled diamond), T (empty circle) and ME (stare); *n* = 3.

The most physicochemical properties that affect the drug's bioavailability are the solubility and dissolution rate of the drug [45]. In vitro dissolution study was investigated to prove the effect of complex formation between sulfonatocalix[4]naphthalene and ME towards enhancing ME solubility and dissolution performance. In vitro dissolution profile of ME–sulfonatocalix[4]naphthalene complexes were performed in the different media, phosphate buffer of pH 7.4, pH 1.2, and distilled water. A significant difference in the dissolution profiles of the prepared ME–sulfonatocalix[4]naphthalene complexes compared with that of the ME commercial product (Moven^®^ 7.5 Capsules, Amman, Jordan) and ME alone was achieved. The coevaporate product in all media showed more than 90% dissolution of the ME after 15 min. These results indicated that the coevaporate product enhanced the dissolution rate of the drug (as illustrated in Figure 6).

In addition, dissolution study results revealed that the percentage of ME released at pH 1.2 from coevaporate, kneading, physical mixture, commercial ME product, and ME alone within 5 min was 84.7 ± 3.5%, 67.2 ± 2.09%, 50.2 ± 3.49%, and 7.6 ± 1.84%, 1.7 ± 1.17%, respectively. Rapid drug dissolution was obtained from a coevaporate mixture followed by kneaded solid powder. Much greater amounts of ME were dissolved from the prepared solid systems compared with that of the ME commercial products and ME alone. At pH 1.2, the percentage of ME released from commercial ME product was about 10.1 ± 1.83 % after 10 min compared with 93.0 ± 2.09%, 86.6 ± 3.52%, and 70.2 ± 3.51% of ME released from coevaporate, kneading, physical mixture, respectively, after the same time(as illustrated in Figure 6). Thus, proofing the superior role of sulfonatocalix[4]naphthalene in enhancing the dissolution of ME. Higher dissolution is also responsible for high absorption, bioavailability, and accordingly rapid and faster action, which is recommended for different types of pain.

The calculated relative dissolution rate (RDR_10_) during the first 10 min and calculated dissolution efficiency (ED_25_) after 25 min [58] for the different prepared soild systems and commercial ME product in different media were calculated and presented in Table 2. Coevaporate product has the highest relative dissolution rate (RDR_10_) after 10 min compared with that of other methods in the investigated media. On the other hand, distilled water medium showed the highest RDR_10_ for the coevaporate, kneading, physical mixture, and commercial ME compared with both pH 7.4 and 1.2. This result might be attributed to the very low aqueous solubility of the drug in distilled water compared with other media, as depicted in Table 2. In addition, coevaporate system showed the highest ED_25_ with average values of 20.86 ± 0.93, 19.00 ± 0.35, 18.76 ± 0.26 at pH 7.4, pH 1.2, and in distilled water, respectively, compared with that of kneaded, physical mixture, commercial product, and ME untreated powder, as illustrated in Table 2. Also, distilled water medium showed the highest increasing fold in dissolution rate and the highest RDR_10_ compared with that of the other used media, pH 7.4 and pH 1.2 followed by pH 1.2. Coevaporate system showed the highest increasing fold and RDR_10_ in distilled water medium, with average values of 65.28 and 90.07, respectively, compared with that of kneaded and physical mixture, commercial product, and untreated ME powder, as illustrated in Table 2. ME showed enhanced dissolution at pH 1.2 compared with pH 7.4, which mimics the gastric pH. Thus, will be suitable for performing rapid action and faster onset. These results proofed an improvement of the dissolution rate of the ME due to the reduction in crystallinity or complete amorphization of the drug, while formation complexes with sulfonatocalix[4]naphthalene, as confirmed by previous studies. Also, these results are in a good agreement with other researchers who showed that the coevaporate product of the ME-β-cyclodexrine complexes has the highest rate of dissolution compared with that of a kneaded and physical mixture, which confirmed our finding [59,60].

According to the results, the coevaporate product showed the highest dissolution rate, and the physical mixture shows the lowest dissolution rate at pH 1.2. These results indicated that the degree of improvement of the dissolution rate was strongly affected by the preparation technique, dissolution medium, and degree of interaction with sulfonatocalix[4]naphthalene [61]. The enhanced dissolution of ME observed from coevaporate powder could be explained by the conversion of ME from crystalline to amorphous or partial amorphous state. Furthermore, the ME inclusion into the large-deep hydrophobic calixarene cavity due to the intermolecular interaction between the ME and calixarene via hydrogen bonding or/ and *π-π* interaction. Besides, kneaded and physical mixture showed a considerable high dissolution performance due to the effect of the water-soluble carrier, increasing the powder wetting [62] as well as improving the drug solubility, as depicted in the solubility study.

The statistical analysis for the study of the dissolution was performed by using one-way between-subjects ANOVA. The one-way ANOVA was conducted to compare the dissolution rate of the coevaporate, kneaded, physical mixture, and commercial product with the dissolution rate of untreated ME in different media at pH values of 7.4, 1.2, and in distilled water.

The null hypothesis (H_o_) to this test is that there is no significant difference between the means percentage of the drug release from the coevaporate, kneaded, physical mixture, and commercial product and the mean of the untreated ME at *p* ≤ 0.05 in the same medium. The statistical analysis results showed a significant difference between the means dissolution rate of the coevaporate, kneaded, physical mixture, and commercial product compared with the mean of the dissolution untreated ME in the same medium. Depending on the results, the null hypothesis was rejected if the *p*-value ≤ 0.05, and the alternative hypothesis was accepted, which states that there is a difference between the means percentage of the drug release from the coevaporate, kneaded, physical mixture, and commercial product compared with that of the mean of the untreated ME in the same medium at pH 7.4, 1.2, or distilled water. Posthoc tests comparisons using Scheffe test, inducted a significant mean difference between the means percentage of the drug release from the coevaporate, kneaded, physical mixture, and commercial product compared with that of the mean of the untreated ME in the same medium. The results summarize as following: in the medium pH 7.4, the percentage of the drug release increased as the following: coevaporate ≈ kneaded > commercial product > physical mixture > ME alone. In addition, in pH 1.2, the order of increasing the percentage of the drug release was as the following: coevaporate ≈ kneaded > physical mixture > commercial product > ME alone. On the other hand, in distilled water all of them showed higher percentage of the drug release compared with ME alone, and there is no significant comparison between each other methods used.

Also, the statistical analysis one-way between-subjects ANOVA for the dissolutions rate was performed. The null hypothesis (H_o_) to this test is that there is no significant means difference between the percentage of the drug release in the different media at pH 7.4, 1.2, and at distilled water by using the same method (coevaporate, kneaded, physical mixture, commercial product, or ME alone) at *p* ≤ 0.05. The null hypothesis (H_o_) was accepted for coevaporate, kneaded, and physical mixture, which means the percentage of the drug release by coevaporate, kneaded, or physical mixture methods are not affected by the used medium for dissolution. On the other hand, the null hypothesis (H_o_) was rejected for commercial product and ME alone, and the alternative hypothesis was accepted. The posthoc tests comparisons using Scheffe test inducted a significant mean difference between the means percentage of the drug release from the commercial product and untreated ME in the phosphate buffer of pH 7.4 compared with that of other media.

## 4. Conclusions

The aqueous solubility of the ME was enhanced to about 23.99 folds at phosphate buffer of pH 7.4 by complexation with sulfonatocalix[4]naphthalene, forming A_L_ type phase-solubility diagram with a 1:1 stoichiometric ratio. Coevaporate method showed the ability to form an inclusion complex of ME inside the cavity of sulfonatocalix[4]naphthalene compared with kneaded and simple physical mixture. Physicochemical characterization of the prepared solid systems using different techniques such as DCS, TGA, PXRD, and FT-IR proved such interaction. Coevaporate product enhanced the ME dissolution performance in all investigated dissolution media compared to that of physical and kneaded powders. Also, coevaporate product showed the highest increasing dissolution fold and relative rate of dissolution (RDR10) in all investigated media, with average values ranging from 5.4–65.28 folds and 7.3–90.7, respectively. The one-way ANOVA statistical analysis at *p*-value ≤ 0.05 revealed the highest percentage of the ME released from coevaporate product in all investigated media. Interestingly, ME showed a significantly *p* ≤ 0.05 higher dissolution performance from coevaporate system compared to that of the commercial ME product, which is useful for fast control of pain in acute and chronic disorders. Finally, the applicability and importance of sulfonatocalix[4]naphthalene for enhancing the solubility and dissolution of poorly water-soluble drugs which would have a potential improvement in its bioavailability after in vivo administration. Moreover, it could be considered an alternative to some toxic cyclodextrins. Future studies should include manipulation of this promising host carrier for enhancing the solubility of different water-insoluble compounds with an emphasis on in vivo studies.

## Figures and Tables

**Table 1:** Summary results of phase solubility studies of the ME- sulfonatocalix[4]naphthalene.

Media	Type of Phase Solubility Diagram	Stability Constant ± S.D (M^−1^)	Increasing of Solubility (*S_t_*/*S_o_*) ^*^
Phosphate buffer pH 7.4	A_L_	1159.739 ± 0.084	23.993
Distilled water	A_L_	653.256 ± 0.167	5.695
0.1 M HCl pH 1.2	A_L_	555.953 ± 0.021	4.369

* *S_t_* solubility of ME in sulfonatocalix[4]naphthalene solution; *S_o_*, solubility of ME in Water.

**Table 2:** Dissolution Efficiency (ED_25_) and Relative Rate of Dissolution (RDR_10_) in different media for the coevaporation (CO), kneaded (KN), physical mixture (PM), commercial meloxicam, and meloxicam untreated powders. (*n* = 3, ±S.D.).

Methods	ED_25_ ± SD, *n* = 3			Fold Increase			RDR_10_		
	pH 7.4	pH 1.2	D.W	pH 7.4	pH 1.2	D.W	pH 7.4	pH 1.2	D.W
CO	20.86 ± 0.93	19.00 ± 0.35	18.76 ± 0.26	5.38	23.34	65.28	7.31	36.05	90.07
KN	19.73 ± 0.92	19.24 ± 0.68	16.47 ± 0.47	5.09	23.63	57.30	6.76	33.56	82.61
PM	13.24 ± 0.76	14.82 ± 0.72	14.50 ± 0.43	3.42	18.21	50.47	4.37	27.19	72.89
Commercial ME	15.38 ± 0.43	2.74 ± 0.053	17.11 ± 0.47	3.97	3.37	59.55	5.11	3.92	81.95
ME powder	3.88 ± 0.46	0.81 ± 0.29	0.29 ± 0.12	-	-	-	-	-	-

## Data Availability

Not applicable.
